# Perivascular spaces and brain waste clearance systems: relevance for neurodegenerative and cerebrovascular pathology

**DOI:** 10.1007/s00234-021-02718-7

**Published:** 2021-05-21

**Authors:** Kaylene Gouveia-Freitas, António J. Bastos-Leite

**Affiliations:** Faculty of Medicine, University of Porto, Alameda do Professor Hernâni Monteiro, 4200–319, Porto, Portugal

**Keywords:** Brain, Cognitive impairment, “Glymphatic”, Lymphatic, Perivascular spaces, Virchow-Robin spaces

## Abstract

Perivascular spaces (PVS) of the brain, often called Virchow-Robin spaces, comprise fluid, cells and connective tissue, and are externally limited by astrocytic endfeet. PVS are involved in clearing brain waste and belong to the “glymphatic” system and/or the “intramural periarterial drainage” pathway through the basement membranes of the arteries. Related brain waste clearance systems include the blood–brain barrier, scavenger cells, cerebrospinal fluid, perineural lymphatic drainage pathways and the newly characterised meningeal lymphatic vessels. Any functional abnormality of PVS or related clearance systems might lead to accumulation of brain waste. It has been postulated that PVS enlargement can be secondary to accumulation of β-amyloid. Lack of integrity of the vascular wall, microbleeds, cerebral amyloid angiopathy (CAA) and enlarged PVS often occur in the preclinical stages of Alzheimer’s disease, preceding substantial brain atrophy. PVS enlargement in the form of *état criblé* at the basal ganglia has also been considered to reflect focal atrophy, most probably secondary to ischaemic injury, based upon both pathological and imaging arguments. In addition, distinct topographic patterns of enlarged PVS are related to different types of microangiopathy: CAA is linked to enlarged juxtacortical PVS, whereas subjects with vascular risk factors tend to have enlarged PVS in the basal ganglia. Therefore, enlarged PVS are progressively being regarded as a marker of neurodegenerative and cerebrovascular pathology. The present review addresses the evolving concept of PVS and brain waste clearance systems, the potential relevance of their dysfunction to neurodegenerative and cerebrovascular pathology, and potential therapeutic approaches of interest.

## Introduction

Perivascular spaces (PVS) of the brain, often called Virchow-Robin spaces, are generally considered to be expansions containing fluid around small vessels. PVS usually correspond to normal findings (or can be even invisible) in the neuroimaging assessment of healthy individuals, but have the tendency to increase in number and enlarge with the process of ageing [1].

PVS are implicated in the drainage (cf. clearance) of waste products from the brain. Specifically, PVS are part of the paravascular way of clearing brain waste [2] and of other recently proposed systems, such as the so-called “glymphatic” system [3], and/or the “intramural periarterial drainage” (IPAD) pathway through the basement membranes of the arteries [4]. It is also established that PVS are related to the blood–brain barrier (BBB) and contain scavenger cells, which remove brain waste. In addition, PVS are related to the cerebrospinal fluid (CSF) circulation and the perineural lymphatic drainage, and are possibly related to the newly characterised meningeal lymphatic vessels [5–7].

The present review aims at describing the evolving concept of PVS and brain waste clearance systems, possible reasons for dysfunction or enlargement of PVS, and how this dysfunction or enlargement can be associated with—or might play a role in—the progress of several pathological conditions causing cognitive impairment and dementia, such as Alzheimer’s disease (AD), cerebral amyloid angiopathy (CAA) and cerebrovascular pathology. Therapeutic approaches that potentially improve the function of PVS and brain waste clearance systems are also described.

## Perivascular spaces of the brain

Although an elementary definition of “perivascular space” may be that of a space surrounding a blood vessel, this term has been credited to several distinct spaces by different authors over more than one century, leading to extensive controversy as to what PVS actually correspond to.

### Historical perspective and current concepts

In 1843, Durand-Fardel observed multiple small holes within the brain, mainly located at the basal ganglia and white matter. He described this observation as the so-called *état criblé* in the textbook *Traité du Ramollissement du Cerveau*. Durand-Fardel reported the *état criblé* as something with a perforated sieve-like appearance, with or without associated brain tissue changes [8].

In the article *Ueber die Erweiterung kleinerer Gefäfse*, published in the Medical Journal *Archiv für Pathologische Anatomie und Physiologie und für Klinische Medicin* in 1851, Virchow described microscopic spaces between the inner/middle *lamina* (*tunica intima* and *media*) and the outer *lamina* (*tunica adventitia*) of the small brain vessels in direct communication with the subarachnoid space. Virchow did not report pathological findings in the corresponding *laminae*, nor in the adjacent perivascular tissue [9, 10].

In 1859, Robin described similar spaces to those previously proposed by Virchow, also postulating that they did not represent any pathological finding, but Robin’s description differed from the former with respect to the location of the spaces, given that this author considered them as closed channels in the outer *lamina* (*tunica adventitia*) of the vessels [9, 10].

There were other descriptions, currently regarded as having historical interest, corresponding—at least partly—to the so-called PVS. Curiously, in 1865, His used an experimental technique and introduced ink directly into the brain tissue, describing spaces surrounding perforating vessels of the brain and spinal cord, which he considered to be the “lymphatics” of the nervous system. These spaces were later assumed to be artefactual by Woollam and Millen [10].

**Fig. 1:**
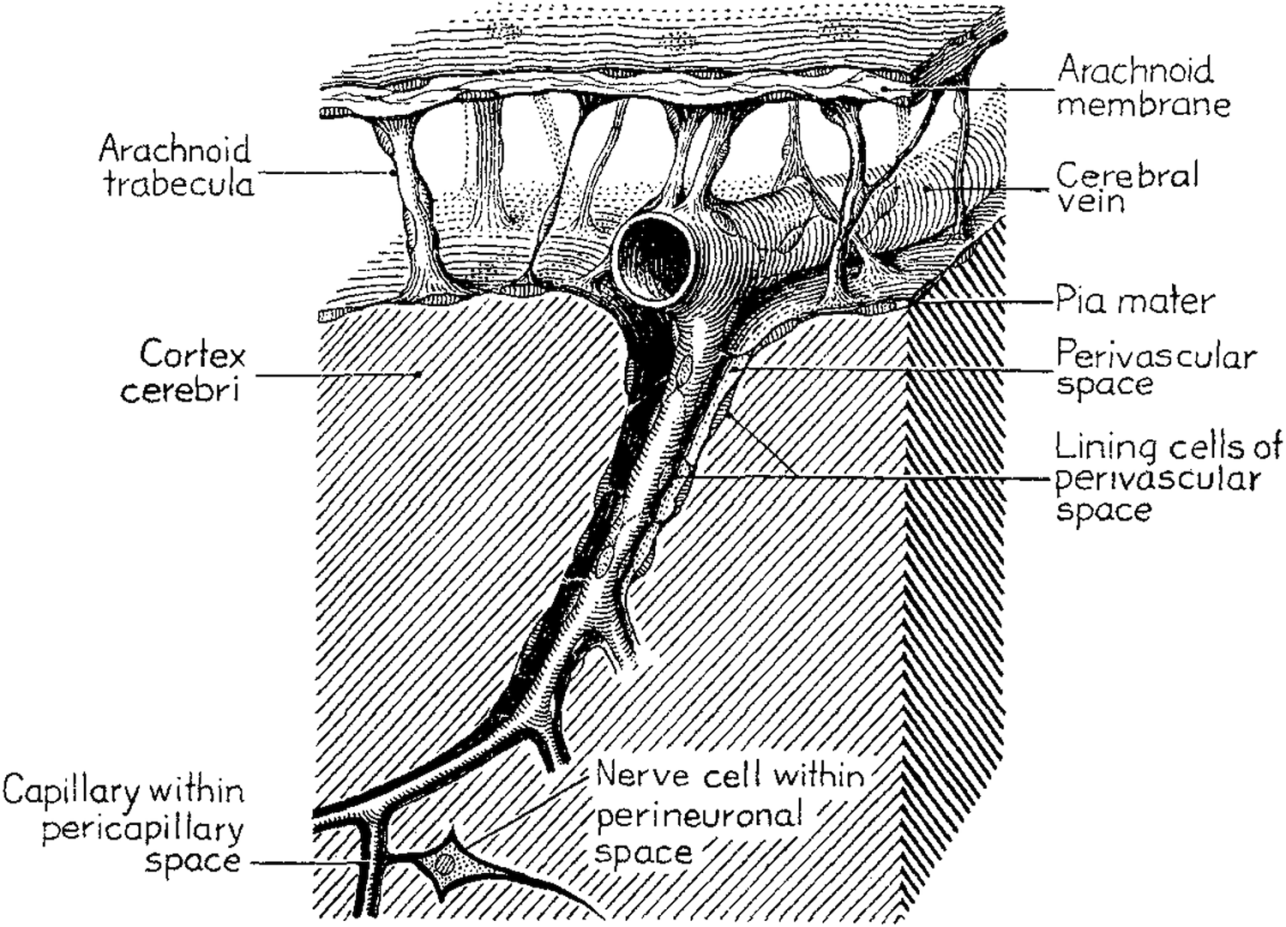
Schematic representation of a perivascular space (PVS) according to Weed et al. (1923). The PVS is represented as communicating borderless with the subarachnoid space, in keeping with the assumption that perivascular spaces were mere expansions of the subarachnoid space into the brain. Reproduced from Weed LH. The absorption of cerebrospinal fluid into the venous system. *American Journal of Anatomy*. 1923; 31(3):191–221 [11]

In 1875, Key and Retzius postulated the existence of funnel-shaped spaces surrounding the perforating blood vessels into the central nervous system (CNS), enclosed between the pia mater and the arachnoid, and containing CSF. These authors denominated such spaces as *piatrichter* (a term combining the words pia and *trichter*—a German word meaning funnel). Key and Retzius assumed that these spaces communicated borderless with the subarachnoid space, in keeping with later descriptions from Weed in 1923 [11] and with the assumption that PVS were mere expansions of the subarachnoid space into the brain (Fig. 1).

**Fig. 2:**
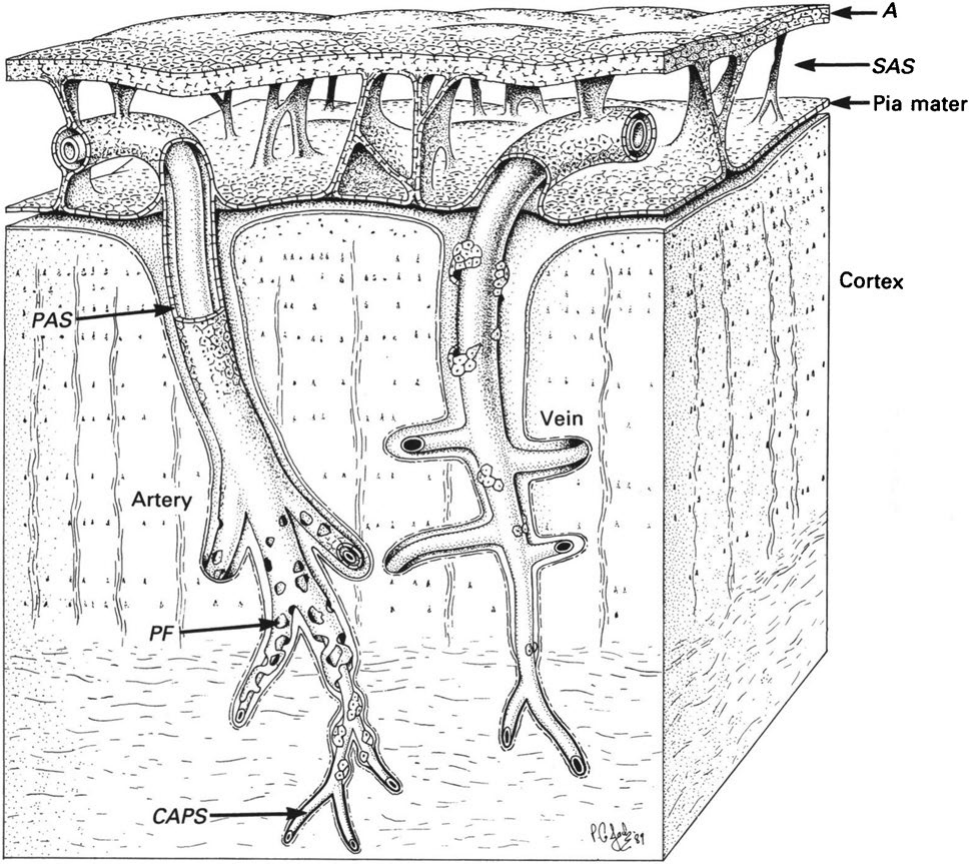
Schematic representation of perivascular spaces (PVS) indirectly communicating with the subarachnoid space (SAS), according to Zhang et al. (1990); the SAS is represented between the arachnoid (A) and pia mater (Pia mater). Please note the existence of a reflected sheath of pia mater onto the surface of the brain at the site of the arterial entry and the venous exit, as well as the existence of leptomeningeal perforations (PF). These perforations increase in size as the vessel wall thickness diminishes, which leads to almost no identifiable pia mater cells at the level of capillaries (CAPS). Reproduced with permission from the Publisher (Wiley) and the authors: Zhang ET, Inman CB, Weller RO. Interrelationships of the pia mater and the perivascular (Virchow-Robin) spaces in the human cerebrum. *Journal of Anatomy*. 1990 Jun; 170:111–23 [13]

In 1982, Krahn [12] carried out a scanning electron microscopy study in cats and rabbits demonstrating that blood vessels were covered by leptomeningeal cells in the subarachnoid space and had a reflected sheath of pia mater onto the surface of the brain at the site of the arterial entry and the venous exit (Fig. 2). It was then postulated that spaces surrounding brain vessels were not in direct continuation with the subarachnoid space. In addition, Zhang et al. [13] have shown that cortical arteries and arterioles of the human brain were surrounded by pia matter cells, but not by spaces. Additionally, in the basal ganglia, arteries were found to be surrounded by a double sheath of leptomeninges (i.e. an inner and an outer layer), whereas no outer layer was described around veins [14].

Recent studies on the microscopic anatomy of the cerebral vascular arterial wall confirmed that there are no PVS around cortical arteries [15, 16]. Sporadic “fenestrations” were described in the pial sheath surrounding small arteries of the brain, as well as “pores or *stomata*” at the CSF-facing leptomeningeal cells, serving as communication points between PVS and the subarachnoid space [13, 17]. This somewhat revives the assumption that, at least in the basal ganglia, there is a communication between the perivascular and subarachnoid spaces indeed. The fact that leptomeningeal openings increase in size (Fig. 2), as the vessel wall thickness diminishes (i.e. as the small arteries progressively enter the brain), leads to no identifiable pia mater cells at the capillary level [13, 18].

**Fig. 3:**
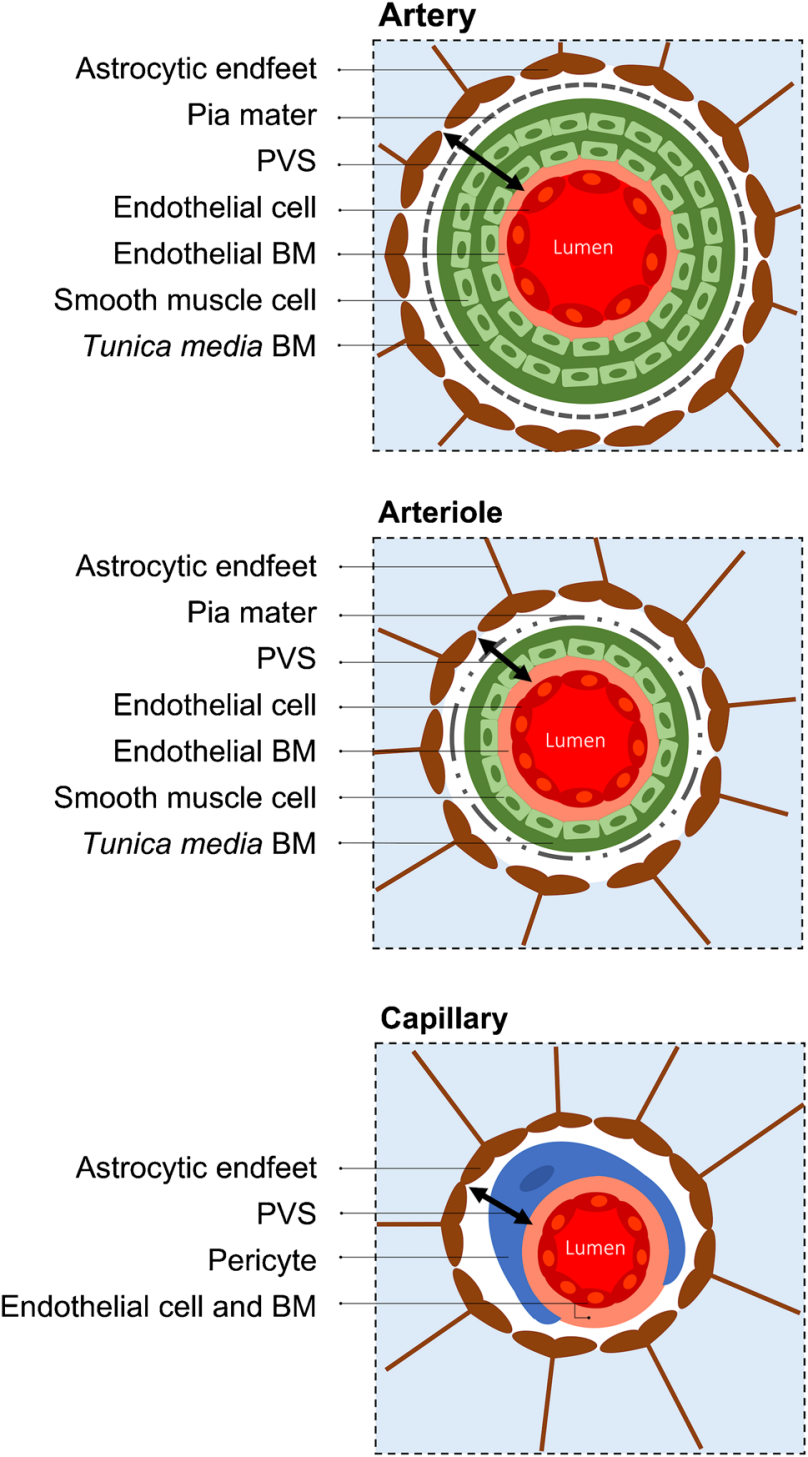
Schematic representation of perivascular spaces (PVS) according to current notions at the level of an artery and an arteriole, and at the capillary level. The arrowheads at each side of the black lines point to the internal (endothelial basement membrane [BM]) and external (“Astrocytic endfeet”) boundaries of the PVS at each level. In general, PVS contain cell populations other than those depicted in the illustration; some are not represented (e.g. scavenger cells other than a “Pericyte”). The artery is surrounded by PVS externally covered by leptomeningeal cells (dashed lines). Fenestrations between leptomeningeal cells covering perforating arteries and arterioles gradually increase in size as the small arteries progressively enter the brain (dashed-dotted lines). At the capillary level, there is no longer an identifiable leptomeningeal covering. The current notion of PVS takes into account any compartment, within or outside the vascular wall, externally limited by the “Astrocytic endfeet” (i.e. glia *limitans*) represented in brown. Any substance entering (or exiting) the PVS from (or to) the brain inevitably crosses the “Astrocytic endfeet”. Likewise, any substance entering (or exiting) the PVS from (or to) the blood inevitably crosses the blood–brain barrier (not specifically represented in the illustration, but corresponding to the vascular endothelial cells, their tight junctions, and the underlying BM)

Current notions regarding the concept of PVS take into account any compartment, within or outside the vascular wall, externally limited by astrocytic endfeet. In other words, PVS may comprise fluid, cells and connective tissue in the form of extracellular matrix (organised enough to be considered as basement membranes surrounding several cell types), and are externally limited by astrocytic endfeet. Furthermore, it is currently accepted that PVS extend deep enough to surround tiny capillary vessels, where they belong to the so-called neurovascular unit and include the following: the pericapillary fluid space between the endothelial cells and the astrocytic endfeet, basement membranes, as well as scavenger cells (Fig. 3) [18, 19].

The aforementioned descriptions helped to clarify the anatomy of spaces surrounding blood vessels in the brain, but they are also on the basis of novel concept proposals underlying brain waste clearance systems described in the following two subsections.

### The “paravascular pathway” and the “glymphatic” system

The so-called “paravascular pathway” was first proposed by Rennels et al. [2] based upon a study using intraventricular infusion of horseradish peroxidase as a tracer in cats and dogs. The circulation of the tracer allowed the authors to postulate the occurrence of an influx of CSF from the subarachnoid space into the PVS—surrounding small perforating arteries and arterioles—followed by influx into the interstitial space of the brain at the capillary level, and then by an efflux of the CSF plus interstitial fluid (ISF) via the perivenous spaces. In spite of not having described the precise anatomical components of this pathway, the authors raised the possibility that the periarterial and perivenous spaces might represent channels of CSF influx and CSF plus ISF efflux, respectively [2].

The concept of “paravascular pathway” was revised on the basis of real-time distribution of tracers, with different molecular weights, injected into the cisternal CSF in mice [3]. The tracers were found to move across the astrocytic endfeet—proposed to have a sieving function—from periarterial and periarteriolar spaces to the interstitial space, where a mixture of CSF with ISF occurs; then, a combination of both fluids would be cleared out at the perivenous spaces. The largest part of the influx was found to occur along small perforating arteries of the basal ganglia and thalamus, and the efflux was found to occur both along the superficial and deep cerebral venous systems. From the perivenous spaces, two terminal pathways of brain waste clearance into the venous blood were proposed: the drainage across the postcapillary vasculature through the BBB, and the clearance via the CSF through the arachnoid *villi* and granulations. An alternative route of brain waste clearance was also proposed along the walls of veins towards the lymphatic cervical lymph nodes. Motive forces supporting a convective bulk flow between the periarterial and perivenous spaces were proposed to be the arterial pulsation and the water transport through aquaporin 4 (AQP4) channels. In short, the combination of the aforementioned anatomical components and mechanisms led to the proposal of the so-called “glymphatic” system [3], a convective pathway of CSF plus ISF recirculation relevant for the removal of brain waste (e.g. β-amyloid) via the venous system.

Although the classical view of the “paravascular pathway” and the “glymphatic” system suggests a size-independent bulk flow of solutes (and water) secondary to arterial pulsation [2, 3, 20, 21], more recent studies have questioned the bulk flow in the interstitial space as well as the facilitating role of water transport by AQP4 channels. As a matter of fact, despite the high hydraulic permeability of PVS [22], it has been claimed that high hydraulic resistance in the interstitial space actually leads to implausibility of any appreciable bulk flow under normal physiological conditions during wakefulness. In other words, such hydraulic resistance of the brain interstitial space might restrict the bulk flow from periarterial to perivenous spaces [18, 22–25]. Therefore, several amendments to the “glymphatic” hypothesis have been proposed in the past few years. The amendments include the role of diffusion [18] and the periarterial pulsation-driven solute transport through dispersion [24]. Diffusion corresponds to a passive process secondary to stochastic Brownian movements [22], whereas dispersion refers to a combined effect of local mixing and diffusion at the periarterial space [24].

Finally, a major criticism of the “glymphatic” system includes the reported absence of PVS around cortical arteries [15, 16]. On theoretical grounds, it seems however plausible that the “glymphatic” system might combine bulk flow and dispersion of solutes at the subcortical periarterial spaces, as well as diffusion at the capillary level and throughout the interstitial space [18].

### The “intramural periarterial drainage” pathway

Following previous proposals since the early-1990s and extensive experimental work on the pattern of β-amyloid deposition in the setting of CAA and AD [26, 27], Weller and Nicoll [28] suggested the periarterial interstitial fluid drainage pathway as being the “lymphatics of the brain”. Later, Carare et al. [29] corroborated this hypothesis and proposed that the brain waste drainage, especially of β-amyloid, would be specifically made via the basement membranes of the capillaries and the basement membranes within the *tunica media* of the arterioles and arteries. This led to a more detailed proposal that such basement membranes could effectively act as the abovementioned “lymphatics of the brain”. Recently, the nomenclature for this brain waste clearance system changed to “intramural periarterial drainage” (IPAD) pathway [4], which is believed to conduct ISF and solutes, but not cells, along the basement membranes of the capillaries, arterioles and arteries towards the cervical lymph nodes via major cerebral arteries in the neck (cf. internal carotid arteries) [30].

Mechanistic insights for this pathway, which is believed to occur in the opposite direction to the arterial blood flow, were proposed also on the basis of a bulk movement of fluid and solutes reaching the basement membranes. Specifically, a so-called contrary (reflection) wave has been proposed to be the underlying motive force. In addition, a valve-like effect—possibly relying upon physical changes in the conformation of basement membranes and biochemical interactions allowing attachment of solutes—was proposed to prevent reflux [31]. A more recently proposed mechanism is vasomotion: a rhythmic variation in the *tonus* of the vascular wall, unrelated to arterial pulsation, which results from spontaneous smooth muscle contraction and relaxation. Actually, vasomotion—a notion first proposed by Jones in 1853—can be the major driving force for IPAD rather than a reflection wave [32].

Further arguments in favour of the IPAD pathway rest upon recent experimental work that did not find unequivocal evidence supporting drainage of brain waste through the veins, as well as on the assumption that tracers injected in the CSF can enter the brain across pial-glial basement membranes, but are then cleared out via distinct pericapillary and periarterial basement membranes [33].

#### Basement membranes

Basement membranes are specialised, laminar, sheaths of extracellular matrix that underlie or surround several cell types, including the vascular endothelium and muscular cells. Basement membranes are mostly formed by the glycoprotein laminin and by collagen type IV. Laminin is believed to initiate the formation of basement membranes, whereas collagen type IV seems to play a role in the corresponding stabilisation and protection from mechanical stresses [34].

Five minutes after injecting fluorescent β-amyloid into the subarachnoid space of mice, Albargothy et al. [33] demonstrated a co-localisation of β-amyloid with α-2 laminin at the pial-glial basement membranes. Twenty-five minutes later (i.e. 30 min after injection), the authors found a co-localisation of β-amyloid with collagen type IV at the basement membranes of smooth muscle cells (cf. *tunica media*) of cortical arteries. This fits comfortably with the interpretation that the IPAD pathway can actually play a role in the elimination of β-amyloid from the brain via the arterial wall [33].

## Related or concomitant brain waste clearance systems

The CNS needs an efficient way of clearing its waste, given that just the brain—although representing 2% of the entire body mass—is responsible for approximately 25% of the global metabolism [35].

Formerly, we have mostly explored how PVS can play a role in clearing brain waste. There are, however, several related or concomitant systems involved in the process, such as the BBB, scavenger cells (e.g. within PVS) and the CSF circulation. Moreover, despite the absence of classical lymphatic vessels in the brain, a sort of lymphatic drainage has been previously subsumed on the basis of a communication between CSF, ISF, brain tissue and the cervical lymph nodes. Furthermore, there are perineural and meningeal lymphatic drainage pathways from the brain to the cervical lymph nodes. These processes play a very significant role to maintain homeostasis, and ultimately contribute to the immune surveillance of the brain [7].

Table 1 summarises the currently proposed brain waste clearance systems. The relative contribution of each of these systems remains unknown. It is, however, suspected that their role might be complementary, because any functional abnormality of a given system may lead to accumulation of brain waste (e.g. β-amyloid) and, therefore, can contribute to the progress of neurodegenerative and cerebrovascular pathology (e.g. AD and CAA).

**Table 1:** Currently proposed brain waste clearance systems

System	Components and clearance location
“Paravascular pathway” and “glymphatic” system	Subarachnoid space → periarterial, periarteriolar, and pericapillary spaces → interstitial space → perivenous spaces → venous blood/cervical lymph nodes (e.g. along the venous walls)
“Intramural periarterial drainage” (IPAD) pathway	Interstitial space → basement membranes of the capillaries in the pericapillary spaces, and basement membranes within the *tunica media* of the arterioles and arteries in the periarteriolar and periarterial spaces → cervical lymph nodes (along the arterial walls)
Blood–brain barrier	Direct vascular transport (e.g. transport of β-amyloid via the LRP1)
Scavenger cells	Intracellular and extracellular brain waste degradation
Cerebrospinal fluid	Arachnoid *villi* and granulations → venous blood; Blood-CSF barrier at the choroid plexuses; Lymphatic pathways
Perineural (cranial and spinal) lymphatic drainage	Subarachnoid space → perineural space (e.g. peri-olfactory lymphatic drainage → cribiform plate → nasal lymphatics) → cervical lymph nodes
Meningeal lymphatic vessels	Subarachnoid space → meningeal lymphatic vessels → cervical lymph nodes

*CSF*, cerebrospinal fluid; *LRP1*, low-density cholesterol receptor-related protein-1

### Blood–brain barrier

The BBB is a very efficient barrier formed by the vascular endothelial cells, their (tight) junctions, and the underlying basement membrane. Put simply, the BBB can ultimately be considered the internal boundary of PVS at the capillary level.

Crucially, it was found that approximately ¾ of the extracellular β-amyloid is directly cleared out of the brain by direct vascular transport across the BBB, mostly via the low-density cholesterol receptor-related protein-1 (LRP1), a process believed to be dependent on the apolipoprotein E (APOE) isoforms [36, 37].

### Scavenger cells

PVS contain resident and migratory scavenger cells [38]. Apart from being involved in the immune surveillance of the CNS, scavenger cells contribute to the degradation of extracellular and intracellular waste. Resident scavenger cells mostly include microglia—native, long-living, macrophages derived from the yolk sac [38, 39]. Migratory scavenger cells have a peripheral origin (i.e. from the blood), correspond to monocyte-derived macrophages, include perivascular macrophages and act as assistant cells when microglia becomes activated but is no longer able to contribute to homeostasis and CNS protection [38, 40].

Pericytes (a specific cell population surrounding microvessels) have also been proposed to be relevant in the process of uptake of soluble β-amyloid [36]. In fact, the LRP1 contributing for such an uptake process is expressed both at the abluminal side of the BBB and at the surface of pericytes, as recently demonstrated in one experimental study in rats [41]. Therefore, pericytes can be considered as resident scavenger cells within PVS at the capillary level. In the past decade, it also became apparent that pericytes are particularly important for the maintenance of BBB integrity [36, 37].

In 1981, Mato and Ookawara [42] described a population of fluorescent granular perithelial cells in rats, formerly assumed to be macrophages, located within cerebral PVS. These cells were found to be capable of scavenging waste products from the brain and bloodstream. Recently, such a cell population was found to be similar to a novel one discovered in the zebrafish brain and is currently regarded as a sort of mural lymphatic endothelial cell [35, 43, 44]. Although these cells are present individually and do not form vessels in the zebrafish brain, they were found to express lymphatic endothelial markers. Therefore, it is conceivable that such cells have a lymphatic origin indeed, but it is currently uncertain whether their counterpart actually exists in humans [35].

Finally, both intracellular and extracellular waste degradation processes can also be carried out by astrocytes externally limiting PVS at the microvascular level [45].

### Cerebrospinal fluid

It is generally considered that CSF functions as a sink for metabolic brain waste products. In addition, CSF may work in combination with the remainder brain waste clearance systems (e.g. along PVS) as a way of clearing interstitium-derived antigens and immune cells out of the brain [7].

According to the traditional view, CSF is produced in the choroid plexuses, circulates throughout the ventricles, exits across the foramina of Magendie and Luschka to reach the subarachnoid space, and then is absorbed through the arachnoid *villi* and granulations into the blood of the venous sinuses. However, there has been debate regarding the main site of CSF reabsorption [46]. In fact, the blood-CSF barrier at the choroid plexuses and the perineural and meningeal lymphatic pathways are now regarded as relevant exit routes for the CSF [45]. This speaks to the notion that CSF and the lymphatic system have a close functional association.

Furthermore, the so-called Bulat-Klarica-Orešković hypothesis postulates that ISF and CSF are predominately formed by water filtration throughout the walls of the capillaries in the CNS. According to this hypothesis, the formation and absorption of ISF and CSF occur at the capillary level, across the BBB, as a result of osmotic and hydrostatic pressures. In other words, when the capillary hydrostatic pressure is higher than the osmotic counter-pressure, filtration of water occurs, whereas when the capillary osmotic counter-pressure is higher than the hydrostatic pressure, there is reabsorption of water at the capillary wall. Contrary to the traditional view, the Bulat-Klarica-Orešković hypothesis postulates that the choroid plexuses mostly contribute to CSF absorption (not to secretion), although it is acknowledged that a bidirectional exchange of fluid occurs at this level. A permanent exchange of water and solutes between the CSF and brain tissue is also postulated by this hypothesis [47–49], for which PVS certainly play a crucial role.

### Perineural lymphatic drainage

It has long been established that CSF and cerebral ISF drain to deep cervical lymph nodes [50, 51]. In fact, the perineural pathways along the optic and olfactory nerves are regarded as relevant lymphatic routes. The peri-olfactory lymphatic drainage route (i.e. around the olfactory nerve sheath) has been particularly well described in mammals, through the cribiform plate and nasal submucosa, before reaching the lymphatic system [7, 46]. This route allows the passage of T cells and antigen-presenting cells into nasal lymphatics and cervical lymph nodes [52].

### Meningeal lymphatic vessels

In 2015, meningeal lymphatic vessels lining the transverse and the superior sagittal sinuses were described [6], as well as at the skull base and along the middle meningeal arteries in mice [5]. This stunning discovery was also replicated in living humans using magnetic resonance imaging (MRI) [53]. The precise anatomical location of the meningeal lymphatic vessels relative to the meningeal layers is yet to be clarified [54], as well as the anatomical connection between such vessels and the subarachnoid space. Nevertheless, studies in mice demonstrated that meningeal lymphatics are capable of modulating both the influx of CSF and solutes into the brain and the efflux of solutes, CSF and ISF. Therefore, there is a potential connection between meningeal lymphatic vessels and the “glymphatic” system, the impairment of which implicating cognitive decline secondary to β-amyloid accumulation and AD pathology [55].

## Perivascular spaces dysfunction or enlargement: relevance for neurodegenerative and cerebrovascular pathology

The precise pathophysiological mechanisms underlying dysfunction or enlargement of PVS are currently uncertain. Age-related factors leading to microvascular changes in the brain, including atherosclerosis, arteriolosclerosis and elastin dysfunction, reduce the compliance of arteries and arterioles, as well as the amplitude of their pulsations, compromising the role of PVS in clearing brain waste [56–58]. Therefore, a reduction or absence of a strong arterial pulsatile flow may delay the transport of β-amyloid, which is kept for a longer time in PVS, facilitating its attachment and deposition in the corresponding basement membranes. Actually, it has been postulated that PVS dysfunction or enlargement can be secondary to accumulation of β-amyloid [29].

Vascular basement membranes increase in thickness with age due to accumulation of collagen fibres [59]. It is also conceivable that the eventual inability of collagen type IV to repair damage secondary to mechanical stresses might lead to PVS dysfunction or enlargement. As a matter of fact, collagen type IV mutations were found to be associated both with PVS enlargement and a specific type of microangiopathy [60, 61].

Loss of perivascular AQP4 polarisation, which occurs in the ageing brain or as a result of reactive astrogliosis, is also believed to compromise brain waste clearance through the “glymphatic” system [62].

Exposure to high-fat diet or hypercholesterolaemia leads to downregulation of collagen type IV, fibronectin and apolipoprotein E, as well as to upregulation of astrocytic and perivascular macrophage markers. Furthermore, it decreases the number of pericytes and the corresponding ability to clear β-amyloid [63]. Additional vascular risk factors such as hypertension and diabetes, as well as cardiovascular disease, pollution and sedentary lifestyle, can initiate a cascade of events culminating in BBB breakdown and dysfunction [64].

Crucially, BBB breakdown as a result of loss of tight junctions, pericytes and endothelial degeneration leads to accumulation of blood-derived components in the vascular wall and PVS. This promotes PVS dysfunction or enlargement, which leads to lack of removal and subsequent accumulation of β-amyloid in the vascular wall and the brain. BBB breakdown is indeed associated with the progress of cognitive decline [65]. It has also been demonstrated that perivascular clearance dysfunction—essentially secondary to age-related changes in the vascular wall or BBB breakdown—leads to a vicious feed-forward cycle, further driving PVS dysfunction and β-amyloid accumulation both in the vascular wall and in the brain [66]. This contributes to neuronal loss, for which the intracellular accumulation of abnormally phosphorylated tau protein plays a role. Lack of integrity of the vascular wall in association with the occurrence of microbleeds, CAA and enlarged PVS often occurs in the preclinical stages of AD, preceding substantial brain volume loss [37].

Neurodegenerative and cerebrovascular pathology, which often coexist, are the most common causes of cognitive impairment. AD is the most frequent neurodegenerative disease causing dementia. The main histopathological findings of AD include intracellular accumulation of abnormally phosphorylated tau protein (both in the form of neurofibrillary tangles and neuropil threads), extracellular accumulation of β-amyloid (e.g. neuritic plaques), and neuronal loss. AD is the most common cause of cognitive impairment, mostly secondary to grey matter loss. The second most common cause of cognitive impairment is cerebrovascular pathology, which can involve the small or the large vessels, can involve the grey or the white matter, and may have a local or systemic cause [67].

Neurodegenerative and cerebrovascular pathology have a close interrelationship. It is plausible that the latter can contribute to the development of AD. Actually, it is well established that cerebrovascular pathology and late-onset AD share common risk factors. Likewise, cerebrovascular abnormalities can lower the threshold for cognitive impairment or increase its severity in the setting of AD. Moreover, there is a significant proportion of patients with cerebrovascular abnormalities and neuroimaging features of neurodegeneration [68, 69]. Furthermore, CAA typically reflects a specific combination of neurodegenerative and cerebrovascular pathology, as it refers to the deposition of β-amyloid in the vascular wall [67].

There is an increasing body of knowledge supporting that enlarged PVS are associated with dysfunctional brain waste clearance systems. Moreover, enlarged PVS are progressively being regarded as a marker of neurodegenerative and cerebrovascular pathology, such as AD and CAA [1, 70, 71]. Furthermore, enlarged PVS were also proposed to be a marker of BBB dysfunction [72].

### Neuroimaging

One of the first MRI-pathological correlation studies published by Braffman et al. [8] described PVS as isointense foci relative to the CSF on all MRI sequences. In other words, PVS are hypointense on T1-weighted images and T2-weighted fluid-attenuated inversion recovery (FLAIR) images, isointense on proton density (PD)-weighted images and hyperintense on T2-weighted images. They were also described as small round or linear, sharply demarcated, structures enlarged enough to be seen on MRI.

Most of the PVS visible on MRI measure < 2–3 mm in cross-sectional diameter [73, 74] and are often found (even in young subjects) surrounding perforating arteries entering the striatum through the anterior perforated substance. PVS are also frequently found in elderly subjects at the *centrum semiovale* or at the cortical-subcortical transition [1, 75]. Another usual location of PVS is the midbrain [75], near the posterior perforated substance.

The distinction between abnormally enlarged and non-abnormally enlarged PVS can be made on the basis of the corresponding shape rather than size. Abnormally enlarged PVS appear to be ectatic and less regular than those non-abnormally enlarged, especially on images representing PVS in a longitudinal view. In addition, a strategy for the neuroradiologist to correctly recognise enlarged PVS in the setting of a pathological condition can be made by taking into account the appearance of adjacent tissue [9]. A key example here is the observation of “radiating stripes with a signal intensity closer” to the cerebral normal-appearing white matter in metachromatic leukodystrophy (MLD) both on T1- and T2-weighted MRI. This is a crucial imaging finding with diagnostic significance, secondary to the occurrence of metachromatic deposits in macrophages within PVS [76]. MLD is a metabolic and genetic condition (with possible late-onset) that can cause cognitive impairment and psychosis [67]. Likewise, abnormally enlarged PVS occur in several other lysosomal storage diseases (e.g. mucopolysaccharidoses), as well as in the setting of infectious and inflammatory disorders [9, 76].

Enlarged PVS can be assessed either using visual rating scales [71, 77–79], or more sophisticated quantitative neuroimaging approaches on MRI acquired at field strengths up to 3 T. Whereas visual rating is observer-dependent and involves ordinary scales constrained by floor and ceiling effects [80], automatic segmentation approaches might be more precise to detect longitudinal changes. Segmentation-based imaging methods can provide an automatic measure of number, volume, and enlargement of PVS [81, 82]. One study using a deep learning algorithm based on a convolutional neural network regression model to automatically quantify PVS scores on T2-weighted images of the brain found the automatic scores to be more “objective” and “less time consuming” than visual rating scores, but no further image contrasts (other than T2) were used as input images, which might have led to misclassification of lacunar infarcts as PVS [83]. By optimising parameters for multi-modal (multispectral) segmentation using both T1- and T2-weighted MRI as source images, and by combining the ensuing segmented images, estimates of PVS “burden” were found to be strongly correlated with neuroradiological assessments. This enabled both longitudinally and transversally oriented PVS in the *centrum semiovale* to be represented. Although fully automatic methods of the sort are not prone to inter- or intra-rater variability, more work is needed to ascertain PVS classification in subjects with concomitant cerebrovascular abnormalities. In addition, visual editing is likely to be necessary in complex cases, but an accurate segmentation of PVS may facilitate the analysis of their spatial distribution, orientation and density [84]. The combination of T1- and T2-weighted images also serves to enhance PVS contrast by using a dedicated filtering algorithm to remove “non-structured high frequency spatial noise”, which enables to intensify the visibility of PVS, both for visual rating and for automatic quantification [85]. Another multispectral segmentation algorithm combining bias-corrected T1-weighted images with T2-weighted FLAIR images, as well as an approach to correct misclassification of white matter hyperintensities (WMH) and a combination of signal intensity- and morphology-based steps, ensured accurate classification of enlarged PVS. In particular, the morphology-based step of this algorithm, which took into account both length and width, seems to be particularly useful to provide additional insights into the clinical significance of enlarged PVS [86]. Other combinations of image contrasts, including T1- and T2-weighted, PD-weighted, and T2-weighted FLAIR images for multispectral segmentation of enlarged PVS, in association with morphology-based characterisation (i.e. width, volume and linearity), were successfully used as well [87]. It is, however, noteworthy to point out that automatic segmentation of PVS might still not be completely accurate and that the corresponding results should be interpreted with caution. Recommendations for future studies include the acquisition of isotropic images, as well as further harmonisation of imaging protocols and analyses [80].

Although the majority of MRI studies using intrathecal administration of gadolinium-based contrast agent (GBCA)—as a tracer—were carried out in animals, there is some information from human subjects in the setting of concomitant pathological conditions (e.g. aqueduct stenosis, CSF leakage, idiopathic normal pressure hydrocephalus and evaluation of ventriculostomy), but this method may cause gadolinium encephalopathy and has yet to be approved for clinical practice. Therefore, available data is insufficient to enlighten mechanisms of brain waste clearance in the setting of usual or pathological ageing [88, 89]. Human studies using intravenous administration of GBCA to study the “glymphatic” system were also attempted. It was postulated that gadolinium deposition in the basal ganglia might occur via the CSF [89]. In addition, non-invasive MRI tracer studies using inversion pulses (i.e. spatial modulation of magnetisation and arterial spin labelling) and phase-contrast methods were investigated, but it is currently difficult to evaluate ISF dynamics, within the brain, using these methods [89].

Diffusion tensor imaging was applied to analyse diffusivity along the PVS, and a significant correlation was found between higher diffusivity and mini-mental state examination scores, indicating an association between lower water diffusivity (along PVS) and increased severity of neurodegenerative pathology presumably of the Alzheimer type [90].

Additional novel approaches include ultra-high-field MRI, acquired at ≥ 7 T, to characterise PVS at the millimetre scale [91–93]. T2-weighted images acquired at 7 T offer an isotropic spatial resolution (voxel size = 0.4 × 0.4 × 0.4 mm) and an entire brain coverage at a scanning time of approximately 8 min. They enable to increase detectability of PVS, especially in patients with AD, both using a visual rating scale and an automatic segmentation method [92]. Diffusion-weighted imaging at ultra-high field takes advantage of PVS fluid to increase their conspicuity, especially using relatively low *b*-values (i.e. < 1000 s/mm^2^). Nevertheless, there are limitations of ultra-high-field MRI secondary to an increased sensitivity to motion, which causes artefacts (e.g. secondary to physiologic involuntary and/or spontaneous movements, such as heartbeat and respiration). There is also increased magnetic field inhomogeneity causing decrease in the signal-to-noise ratio, especially at the peripheral part of the brain. This limits the ability to detect juxtacortical PVS. In addition, there are safety issues related both to an increased radiofrequency specific absorption rate (SAR) and to a much more limited range of compatible biomedical implants and devices. The use of appropriate coils and parallel imaging may overcome part of these limitations [93].

Finally, magnetic resonance encephalography can also be used to assess the so-called “glymphatic” clearance, using optical flow estimation analysis aimed at quantifying the cardiovascular pulse propagation in the human brain [94].

#### État criblé, lacunar infarcts, white matter hyperintensities and microbleeds

**Fig. 4:**
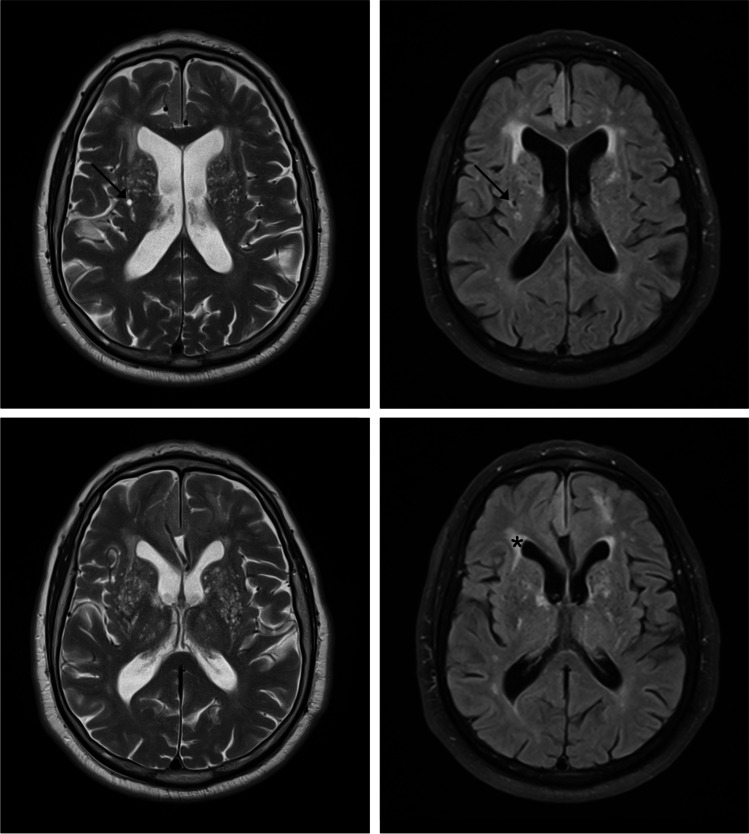
État criblé, lacunar infarct, and white matter hyperintensities. A 77-year-old man with vascular risk factors and cognitive impairment (clinically expressed as lack of executive functioning and apathy) underwent magnetic resonance imaging (MRI). T2-weighted MRI (left column) shows multiple enlarged perivascular spaces (PVS) in the form of *état criblé* at the basal ganglia. Both arrows indicate the concomitance of a small right putaminal lacunar infarct. Please note the irregularly shaped peripheral rim of gliosis hyperintense on T2-weighted fluid-attenuated inversion recovery (FLAIR) image (right arrow) helping to differentiate the lacunar infarct from adjacent PVS. In addition, please note the associated white matter hyperintensities (e.g. asterisk on the bottom right T2-weighted FLAIR image)

Numerous enlarged PVS in the basal ganglia—a condition also referred to as *état criblé*—is a pathological finding [1]. In addition, PVS enlargement in the form of *état criblé* has been considered to reflect focal atrophy based upon both pathological and imaging arguments [95, 96]. It is entirely plausible that such a pattern of focal volume loss, around deep small vessels, may be secondary to ischaemic injury. As a matter of fact, in patients with *état criblé*, it can be difficult to rule out associated focal ischaemic lesions, such as lacunar infarcts (Fig. 4). In 1901, Pierre Marie proposed *état lacunaire* as a distinct entity corresponding to multiple deep lacunar infarcts. Meanwhile, the term *état lacunaire* has been incorporated within the so-called subcortical ischaemic vascular dementia [97]. Although lacunar infarcts are also hypointense on T1-weighted images, they usually have a more irregular shape than PVS and a peripheral rim of gliosis (Fig. 4) hyperintense on T2-weighted FLAIR images [1].

*État criblé* is frequently associated with white matter changes (Fig. 4). In fact, Maclullich et al. [79] postulated an association among enlarged PVS, diffuse confluent WMH and cognitive impairment. Diffuse confluent WMH on T2-weighted images are generally considered to be a neuroimaging marker of ischaemic small vessel disease in elderly subjects associated with considerable hypoperfusion [98].

Microbleeds, also known as haemorrhagic lacunes, are hypointense foci (< 5 mm) on gradient-echo T2*-weighted images. Susceptibility-weighted imaging is currently the preferred MRI sequence for their detection [99]. Microbleeds secondary to vascular risk factors, such as hypertension, are usually found in the basal ganglia, the thalamus and the posterior fossa. Peripheral microbleeds at the cortical-subcortical transition occur in CAA [100] and in other specific types of microangiopathy. In any of those circumstances, the existence of microbleeds is associated with loss of integrity of the vascular wall [101].

#### Cerebral amyloid angiopathy

**Fig. 5:**
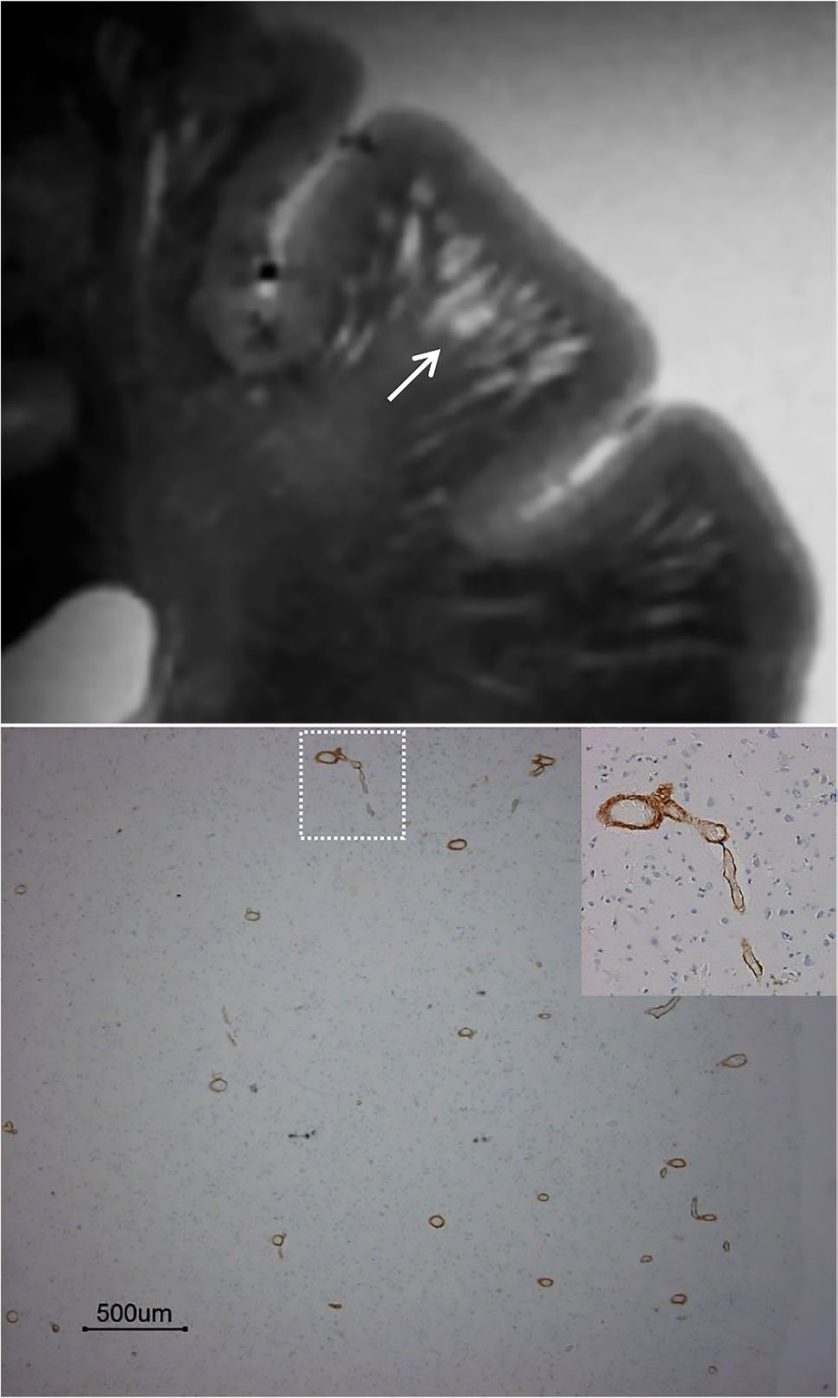
Enlarged juxtacortical perivascular spaces (PVS). Post-mortem T2-weighted magnetic resonance image acquired at 7 T (top) showing sharply demarcated juxtacortical hyperintensities corresponding to “many severely enlarged PVS” (arrow) in a patient with pathologically confirmed cerebral amyloid angiopathy (bottom). Please note the absence of PVS at the cortical level. Modified and reproduced with permission from the Publisher (SAGE) and the authors: van Veluw SJ, Biessels GJ, Bouvy WH, Spliet WG, Zwanenburg JJ, Luijten PR, Macklin EA, Rozemuller AJ, Gurol ME, Greenberg SM, Viswanathan A, Martinez-Ramirez S. Cerebral amyloid angiopathy severity is linked to dilation of juxtacortical perivascular spaces. *Journal of Cerebral Blood Flow and Metabolism*. 2016; 36(3):576–580 [91]

Neuroimaging evidence of CAA occurs in a substantial proportion of patients with AD, even in familial cases without vascular risk factors [102]. Enlargement of juxtacortical PVS has been linked to CAA (Fig. 5). This was attributed to accumulation of β-amyloid and to impairment in the corresponding process of drainage [91], secondary to neurodegenerative pathology of the Alzheimer type. Furthermore, distinct topographic patterns of enlarged PVS are related to different types of microangiopathy. Patients with CAA have enlarged juxtacortical PVS along with peripheral haemorrhages, microbleeds or siderosis. Subjects with vascular risk factors (e.g. hypertension) tend to have enlarged PVS in the basal ganglia, as well as a more severe degree of WMH [103].

## Potential therapeutic approaches of interest

Preventing or minimising the impact of modifiable vascular risk factors (e.g. hypertension, diabetes and hypercholesterolaemia) can help to preserve the integrity and robustness of the vascular wall, and promote an efficient perivascular clearance of brain waste. Accordingly, vasoactive drugs (e.g. inhibitors of phosphodiesterases) can improve perivascular drainage. For instance, the positive chronotropic, elasticity-promoting, arterial pulse duration time increase and vasodilatory effects of cilostazol have been proposed to promote the efficiency of perivascular drainage [104]. Likewise, other inhibitors of phosphodiesterases (e.g. caffeine and sildenafil) were found to have similar effects, but further experimental studies are needed to confirm their efficiency [7]. These therapeutic approaches have the potential to enhance both the “glymphatic” system and/or the IPAD pathway.

Certain therapeutic approaches were specifically devised to enhance the efficiency of the “glymphatic” system by increasing the production of ISF or expanding the brain extracellular space. The increase in ISF production can be attained at the BBB level (e.g. secondary to brain-derived arginine vasopressin at the abluminal secretory surface of the endothelium) [105]. Natural sleep (or anaesthesia) was found to be associated with a 60% increase in the brain interstitial space of mice. Considering the aforementioned high hydraulic resistance in the brain interstitial space during wakefulness, sleep might increase the convective flow of CSF plus ISF and, therefore, the efficiency of the “glymphatic” system [106]. Sleep is also known to be associated with increased CSF production. After administration of adrenergic antagonists in mice during wakefulness, CSF influx and the extracellular space were found to increase to levels comparable to those observed during sleep or anaesthesia. Therefore, a decrease in adrenergic signalling results in expansion (and decreased resistance) of the brain extracellular space. Actually, sleep—a state of decreased adrenergic tone—is associated with an increased rate of β-amyloid clearance [106]. Curiously, a single night of sleep deprivation results in accumulation of β-amyloid in the human hippocampus and thalamus [107]. Sleep quality improvement has been proposed to reduce the risk of AD [108], and a lateral position of the head during sleep seems to promote the efficiency of the “glymphatic” system, according to results of one study carried out in anesthetised rats [109]. In general, an adequate sleep time, a consolidated sleep pattern, the avoidance of sleep fragmentation, and treatment of sleep disorders may constitute general future recommendations to avoid neurodegeneration [108].

Studies using a rat model of traumatic brain injury found that statins reduce reactive astrogliosis [110] and, possibly, the accumulation of tau protein leading to neurodegeneration. This assumption led to the speculation that statins could improve the “glymphatic” system efficiency by preserving the perivascular polarisation of AQP4 channels [111]. Physical exercise has also been shown to promote “glymphatic” clearance of β-amyloid in aged mice, secondary to improved AQP4 expression and polarisation, as well as to reduced microglia activation and astrogliosis [112].

Therapeutic approaches aimed at eliminating β-amyloid deposits in the vascular wall and facilitating the function of PVS may be achieved by targeting brain waste clearance systems other than the “glymphatic” one. Studies in mouse models have shown the possibility to enhance β-amyloid clearance, using focused ultrasound, via the BBB. Such enhanced β-amyloid clearance was found to be associated with improvement of memory and behavioural measures [113, 114]. Targeting scavenger cells also revealed promising results. For instance, the activation of perivascular macrophages might constitute a valid therapeutic strategy to clear vascular amyloid and enable further brain waste clearance through the PVS [115]. Likewise, targeting other scavenger cells, such as the pericytes, by increasing the activity of the LRP1/APOE clearance system, has potential therapeutic effects. Specifically, injecting pericytes derived from stem cells of APOE ε3 carriers (i.e. subjects at low risk of developing neurodegeneration) or using genome-editing technologies in pericytes of APOE ε4 carriers (i.e. subjects at high risk of developing neurodegeneration) is an approach to consider, so that pericytes with enhanced ability of clearing β-amyloid can be generated [41].

Strategies to facilitate perivascular clearance of soluble β-amyloid in combination with novel anti-β-amyloid immunotherapies yield a potential therapeutic avenue for both AD and CAA, but the major challenge will be to promote successful removal of insoluble β-amyloid plaques from the brain—as demonstrated in transgenic mouse models after immunisation [116] and in subsequent clinical trials—without substantial entrapment of β-amyloid in the vascular wall or the PVS, so that CAA-related inflammation or other ominous side effects attributable to autoimmune responses (e.g. T-lymphocyte meningoencephalitis) can be avoided [117–119].

Finally, computational models simulating biological and pathophysiological processes not only represent a way of understanding how brain waste clearance systems normally work or how they fail under neurodegeneration but also serve to understand how effective novel therapeutic approaches can be to counteract their failure [120, 121].

## Conclusion

The anatomical concept and terminology of PVS have evolved over more than one century. Although a certain degree of PVS enlargement is often considered to be indicative of normal ageing, dysfunctional and/or enlarged PVS are progressively being regarded as a marker of both neurodegenerative and cerebrovascular pathology.

Dysfunctional and enlarged PVS can be associated with impaired efficiency in removing β-amyloid from the brain. The accumulation of β-amyloid is currently believed to have a major role in the progress of AD and CAA. Therefore, a better understanding of factors involved in the perivascular transport of solutes from the brain is crucial, given that novel therapeutic approaches improving β-amyloid elimination might prevent the development of AD and CAA. This is important, as the pre-symptomatic neuropathological changes start in individuals years or even decades before the clinical manifestation of AD [122].

Insights into PVS function and dysfunction have themselves raised the interest in novel therapeutic approaches aimed at modifying the natural history of neurodegenerative and cerebrovascular pathology. Although the understanding of brain waste clearance systems is improving, current knowledge about their interaction is still insufficient. A key example here is the assumption that the “glymphatic” system and the IPAD pathway correspond to two different (or independent) proposals to explain brain waste clearance. It is, however, relevant to point out that each of the proposals resulted from distinct research methodologies. Specifically, the location of tracer injection and the timing of the corresponding movement evaluation differed among several experiments leading to each of the proposals [3, 4, 29]. Therefore, more work is needed to clarify to what extent both proposals can correspond to a common underlying brain waste clearance system or represent “two faces of the same coin”. An alternative possibility is to explore whether the role of each of these two systems can be the transport and distribution (along PVS) of solutes (e.g. β-amyloid) cleared out through further concomitant ways.
